# An effective live-attenuated Zika vaccine candidate with a modified 5′ untranslated region

**DOI:** 10.1038/s41541-023-00650-w

**Published:** 2023-04-01

**Authors:** Farzana Nazneen, E. Ashley Thompson, Claire Blackwell, Jonathan S. Bai, Faqing Huang, Fengwei Bai

**Affiliations:** 1 Cell and Molecular Biology Program, Center for Molecular and Cellular Biosciences, The University of Southern Mississippi, Hattiesburg, MS 39406 USA; 2 Department of Chemistry and Biochemistry, University of Texas at Dallas, Richardson, TX 75080 USA; 3 Chemistry and Biochemistry Program, Center for Molecular and Cellular Biosciences, The University of Southern Mississippi, Hattiesburg, MS 39406 USA

**Keywords:** Live attenuated vaccines, Infectious diseases

## Abstract

Zika virus (ZIKV) is a mosquito-transmitted flavivirus that has caused devastating congenital Zika syndrome (CZS), including microcephaly, congenital malformation, and fetal demise in human newborns in recent epidemics. ZIKV infection can also cause Guillain-Barré syndrome (GBS) and meningoencephalitis in adults. Despite intensive research in recent years, there are no approved vaccines or antiviral therapeutics against CZS and adult Zika diseases. In this report, we developed a novel live-attenuated ZIKV strain (named Z7) by inserting 50 RNA nucleotides (nt) into the 5′ untranslated region (UTR) of a pre-epidemic ZIKV Cambodian strain, FSS13025. We used this particular ZIKV strain as it is attenuated in neurovirulence, immune antagonism, and mosquito infectivity compared with the American epidemic isolates. Our data demonstrate that Z7 replicates efficiently and produces high titers without causing apparent cytopathic effects (CPE) in Vero cells or losing the insert sequence, even after ten passages. Significantly, Z7 induces robust humoral and cellular immune responses that completely prevent viremia after a challenge with a high dose of an American epidemic ZIKV strain PRVABC59 infection in type I interferon (IFN) receptor A deficient (*Ifnar1*^−/−^) mice. Moreover, adoptive transfer of plasma collected from Z7 immunized mice protects *Ifnar1*^−/−^ mice from ZIKV (strain PRVABC59) infection. These results suggest that modifying the ZIKV 5′ UTR is a novel strategy to develop live-attenuated vaccine candidates for ZIKV and potentially for other flaviviruses.

## Introduction

Zika virus (ZIKV), a mosquito-transmitted flavivirus, was originally isolated in 1947 in Africa, but it had not caused severe human disease until 2007^1,2^. In recent years, ZIKV has been shown to cause congenital Zika syndrome (CZS) in offsprings born to infected pregnant women, non-human primates (NHP), and mice^3–7^. Our research results in mice, along with clinical evidence in humans, have also shown that even a mild congenital ZIKV infection may result in postnatal deficits in mouse and human newborns, even without apparent defects at birth^8,9^. In human adults, ZIKV infections may cause Guillain-Barré syndrome (GBS), an autoimmune disease caused by the immune system attacking the peripheral nerves, leading to a rapid onset of muscle weakness and even paralysis^10,11^. ZIKV is primarily transmitted to humans by the *Aedes* species of mosquito^12,13^, but it can also be acquired through sexual contact, especially from male to female transmission^14,15^. In February 2016, the World Health Organization declared the ZIKV epidemic a public health emergency of international concern due to its explosive outbreaks and significant health concerns^16^. Despite intensive efforts to develop antiviral therapeutics and vaccines, there is still no approved vaccine. Although the number of human Zika cases has dropped since 2017, ZIKV will likely become endemic following the course of other arboviral diseases that have accompanied the invasive *Aedes* mosquitoes to the Western hemisphere^17^. Indeed, it was recently reported that ZIKV has silently spread to almost all parts of India and caused significant morbidity in 2021^18^. Thus, the risk of ZIKV transmission continues, and a vaccine that can prevent CZS and GBS remains urgently needed^17,19,20^.

**Fig. 1:**
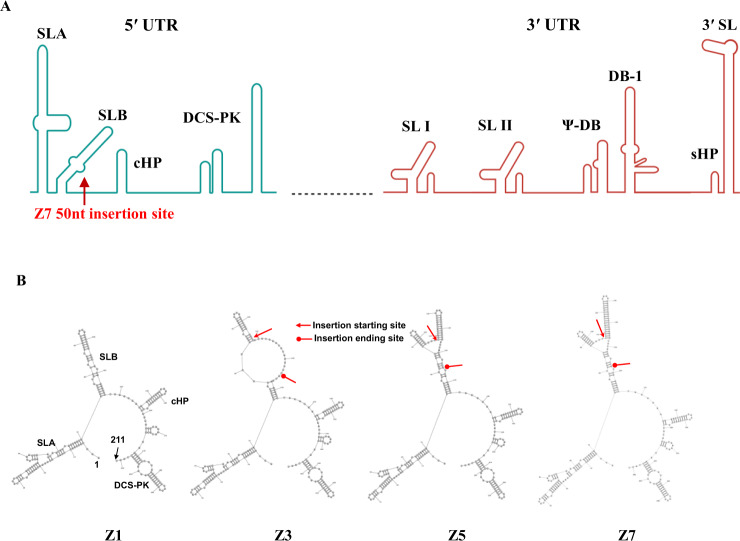
Generation of mutant ZIKV by modifying 5′ UTR. **A** The insertion site of the GC-rich nucleotides in the 5′ UTR of the ZIKV genome^73^. The illustration was created with BioRender. **B** The predicted secondary structures of Z1 (WT, no insert), Z3 (18-nt insert), Z5 (38-nt insert), and Z7 (50-nt insert) by RNAFold.

Flaviviruses have a linear, positive-sense, single-stranded RNA genome, which is comprised of a 5′ untranslated region (UTR), an open reading frame that translates into a polyprotein, and a 3′ UTR^21–23^. The polyprotein is cleaved by the cellular and viral proteases into 3 structural (capsid [C], pre-membrane [PrM/M], and envelope [E]) and 7 non-structural proteins (NS1, NS2A, NS2B, NS3, NS4A, NS4B, and NS5)^21,24^. The ZIKV 5′ UTR is 106-nt, and the 3′ UTR is 428-nt in length^25^. The 5′ UTR consists of an m7GpppAmpN1 cap structure and the conserved stem loops A and B (SLA and SLB, Fig. 1A)^26,27^. The cap structure in flavivirus genomes is important for cap-dependent translation and protection from cellular 5′-3′ exonucleases^26,28^. While SLA serves as a promoter for the viral RNA-dependent RNA polymerase (RdRp) NS5, SLB facilitates replication by cyclizing the RNA genome via the formation of a 5′-3′ complementary structure with the 3′ UTR^26,29,30^. A recent study also found that the ZIKV 5′ UTR pairs with the E protein coding region to form a 5′-E complementary structure, which may play an important role in viral replication or translation regulation. Other conserved RNA structural elements, such as the C coding region hairpin (cHP) and downstream of 5′ cyclization sequence pseudoknot (DCS-PK), were also identified at the 5′ end of the ZIKV genome^26^. Although the 5′ UTR sequences are variable among flaviviruses, the RNA structure is mostly conserved and is essential for genome cyclization, viral RNA synthesis, translation, and viral fitness. The 3′ UTR of ZIKV folds into three domains which are highly structured with regions conserved among flaviviruses (Fig. 1A)^25,26^. Interacting with the 5′ UTR, the 3′ UTR plays a critical role for viral replication^26^.

Most ZIKV vaccine development strategies target the prM-E, the non-structural proteins, and the 3′ UTR, but none focus on the 5′ UTR, which is essential for both RdRp recognition and eIF4E initiation of ribosomal translation. In eukaryotic translation, ribosomes scan, beginning from the methylated cap, for an AUG start codon and stall at areas with stable secondary structures, such as stem-loops, as the ribosomes have to “melt” these structures to continue down the RNA strand^31^. A previous report showed that by adding various lengths of stem loops in eukaryotic cells, translation was slowed due to the increased length of the GC-rich structures^32^. While this 5′ UTR insertion method has been used to attenuate translation in different eukaryotic systems^33,34^, it has not been applied to viral research previously. We hypothesized that modification of the 5′ UTR of the ZIKV genome by inserting a GC-rich sequence would introduce an additional RNA structure that might attenuate circularization and slow down the rate of viral RNA replication and protein translation, thus creating attenuated viruses. In this report, we developed a live attenuated ZIKV vaccine candidate, named Z7, by introducing a GC-rich sequence into the 5′ UTR region of the pre-epidemic ZIKV Cambodian strain, FSS13025. The attenuated ZIKV showed great potential as a novel vaccine candidate by inducing robust antibody and T-cell-mediated immunity against the American epidemic ZIKV (strain PRVABC59) infection in a mouse model.

## Results

### Generation of the mutant ZIKV by modifying the 5′ UTR region

**Fig. 2:**
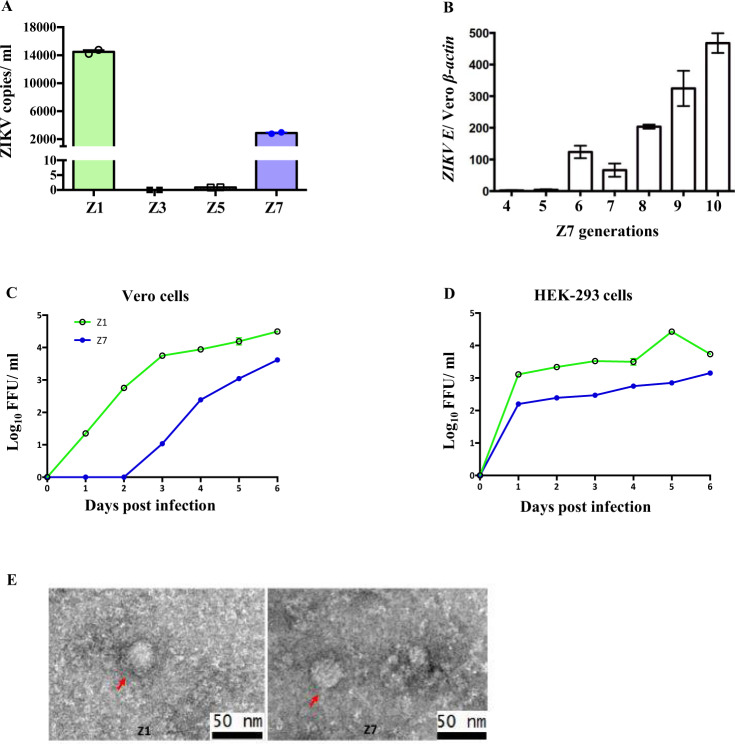
Characterization of Z7 in vitro. **A** Quantification of ZIKV copies of 5′ UTR modified (Z3, Z5, Z7) or unmodified (Z1) plasmids by qRT-PCR. **B** Quantification of *ZIKV E* in Z7 for several generations (G4 to G10) by qRT-PCR. Data were presented as the ratio of *ZIKV E* to Vero *β-actin*. **C** The in vitro growth curves of Vero cell-generated Z1 and Z7 (G10). Vero cells were infected with 0.1 MOI of Z1 or Z7 (G10), and the virus titers in the supernatants were determined by FFA. **D** The in vitro growth curves of HEK-293 cell-generated Z1 and Z7 (G11). HEK-293 cells were infected with 0.01 MOI of Z1 or Z7 (G11), and the virus titers in the supernatants were determined by FFA. **E** TEM images of Z1 and Z7 (G9). **A**–**D** Data were presented as mean ± s.e.m.

To test if a modification of the 5′ UTR affects ZIKV infectivity, we respectively inserted 18, 38, or 50 nt GC-rich DNA sequences (Table 1) at the end of the SLB region and before the start codon (ATG) in the plasmids containing the ZIKV genome (Cambodian strain, FSS13025) (Fig. 1A) by our established cloning method^35^. We chose the ZIKV Cambodian strain as the cloning backbone because it is attenuated in neurovirulence, immune antagonism, and mosquito infectivity compared with the American epidemic isolates^1,2,36^. The predicted 5′ UTR ZIKV RNA structures showed that the 38 and 50-nt inserts resulted in an additional hairpin structure in the SLB, while the SLA, cHP, and DCS-PK structures remained intact (Fig. 1B, Z5 and Z7, respectively). In contrast, instead of forming a new hairpin structure, the insertion of an 18-nt insert resulted in a large loop in the SLB hairpin structure (Fig. 1B, Z3). These modified and unmodified control plasmids were transfected into Vero cells. The rescued ZIKVs (Z1 = WT [no insert]; Z3 = 18-nt; Z5 = 38-nt; and Z7 = 50-nt insert) in the cell culture media were collected on D3 post-transfection. The media containing the first generation (G1) of ZIKVs were used to infect fresh Vero cells to evaluate the viral genome replication using quantitative reverse transcription polymerase chain reaction (qRT-PCR). The qRT-PCR results suggested that insertion of 18-nt (Z3) could not generate live viruses (Fig. 2A). The 38-nt insertion (Z5) resulted in low but detectable *ZIKV E* copies, while the 50-nt insertion (Z7) produced a much higher level of *ZIKV E* copies than Z5 (Fig. 2A).

**Table 1:** The insert RNA sequences.

Name	Z1 (WT)	Z3	Z5	Z7
Insert RNA sequences	None	CGUACGAGCGCAGGUGC	CGACCACGGCCGGAAACGGCCGUGGUCGCGCAGGUGCC	CGUUCCAACCACUGACUCGAAAGAGUCAGUGGUUGGAACGCGCAGGUGCC

Since Z7 could generate higher titers than Z5, we focused on Z7 in this project. We continuously passed Z7 in Vero cells (an approved cell line for vaccine production^37^) for 11 generations (5 days/generation). Interestingly, we found that titers of Z7 gradually increased through the continuous passaging in Vero cells, indicating Z7 may have adapted fitness (Fig. 2B). Next, we determined the growth kinetics of Z7 and Z1 by focus forming assay (FFA) in both Vero and Human Embryonic Kidney (HEK) 293 cells. Compared to Z1 (WT), Z7 was able to grow efficiently in both cell lines, albeit with slightly lower growth rates (Fig. 2C, D). To visualize these plasmid-generated ZIKV particles, we examined Z1 and Z7 viral stocks under a Transmission Electron Microscope (TEM) with negative staining. The TEM images confirmed that Z1 and Z7 exhibited the typical “golf ball” appearance and size (~40 nm) of ZIKV^38^ (Fig. 2E). There is a reasonable concern that the insert may be self-deleted during the viral replication, and thus possibly reverting to its parent WT strain. To determine if the 50-nt insert remains stable during passaging, we sequenced the whole viral genomes of Z7 (G8 to G10) and Z1 by next-generation sequencing, which confirmed the intact insert without mutation, suggesting the insert was stable in the 5′ UTR region. Interestingly, we found two types of mutations, S1417A (primary) or S1417T (secondary), in the same location of NS2B protein in Z7 (G8 to G10, Supplementary Fig. 1), indicating Z7 might adopt fitness mutations through the passaging. Collectively, these results demonstrate that we have successfully generated Z1 and Z7 with a reverse genetic approach, and the 50-nt insert in Z7 is stable for at least 10 generations.

### Z7 exhibits attenuated infectivity in vitro and in *Ifnar1*^−/−^ mice

**Fig. 3:**
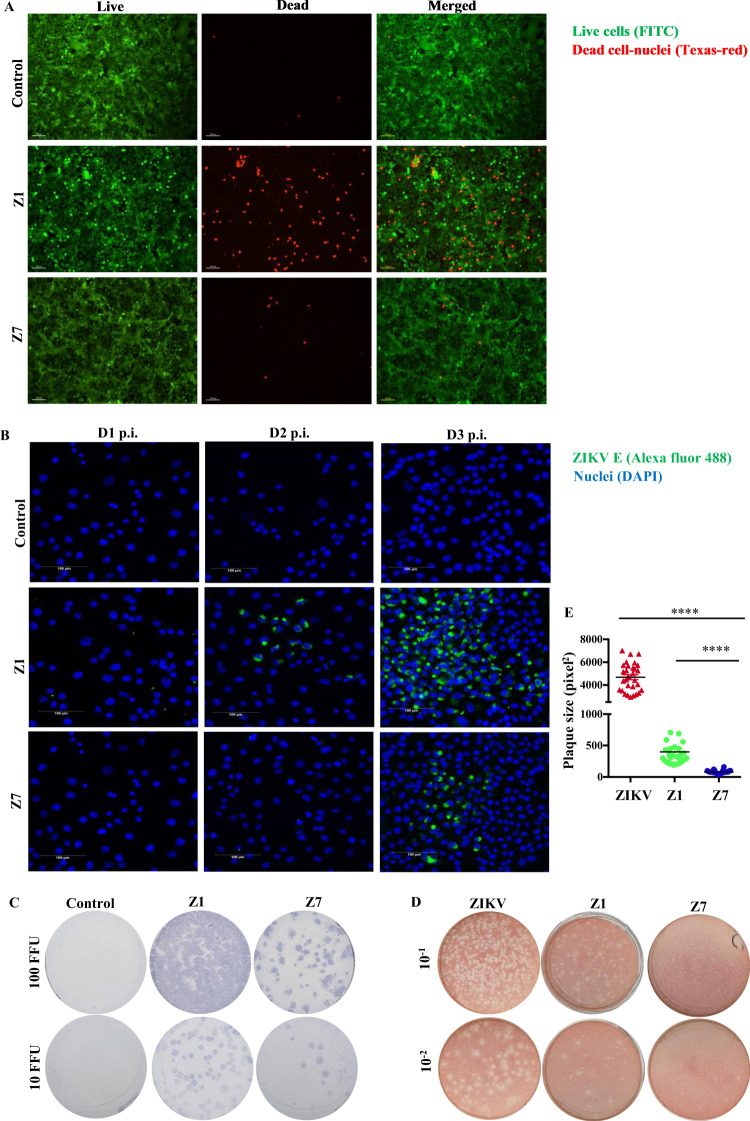
Z7 exhibits attenuated infectivity in vitro. **A** The cytopathic effect (CPE) of Z1 and Z7 on Vero cells. Vero cells were inoculated with Z1, Z7 (G11), or PBS as a control, and incubated for 3 days. The cells were stained with LIVE/DEAD Cell Imaging Kit, which stained the live cells with FITC as green and the dead cell nuclei with Texas-red as red. The images were taken at 10× magnification (scale bar = 100 µm). **B** The IFA for ZIKV E protein expression. Vero cells were infected with 0.1 MOI of Z1, Z7 (G11), or PBS as control and incubated for 3 days. The ZIKV E protein was probed with 4G2 antibody, followed by goat anti-mouse IgG conjugated with Alexa Fluor 488 (green). The cell nuclei were stained with 4′,6-diamidino-2-phenylindole (DAPI, blue). The images were taken at 20× magnification (scale bar = 100 µm). **C** The immunostaining foci of Z1 and Z7. Vero cells were infected with 10 or 100 FFU of Z1, Z7 (G11), or PBS as control and incubated for 3 days (Z1) or 4 days (Z7). The cells were probed with 4G2 antibody and then goat anti-mouse IgG-HRP as a secondary antibody. The Immuno-positive foci were developed with TrueBlue peroxidase substrate. **D** Plaque morphology of ZIKV (strain PRVABC59), Z1, and Z7. Vero cell monolayer was infected with 100 µl of serially diluted ZIKV (strain PRVABC59), Z1 or Z7 (G11) and incubated for 5 days. **E** Plaque sizes of ZIKV (strain PRVABC59), Z1, and Z7 on D5 p.i. Data were compared with a two-tailed Student’s *t*-test and presented as mean ± s.e.m. with ****p < 0.0001.

To determine if Z7 has attenuated pathogenicity compared to Z1, we performed cytopathic effect (CPE) assay, immunofluorescence assay (IFA), FFA, and plaque-forming assay. For the CPE assay, we inoculated Vero cells with 0.1 MOI of Z1, Z7 (G11), or PBS as control and examined the CPE on the cells under a Leica M165 FC microscope. The results showed that Z1 caused apparent CPE effects in Vero cells starting on D3 post infection (p.i.) by killing more cells than Z7 (Fig. 3A), while Z7 did not cause noticeable CPE for 4 days compared to the control (D4 p.i., Supplementary Fig. 2). To determine if Z7 is less infective in vitro, we measured the expression of ZIKV E protein in Vero cells by IFA, in which Vero cells were infected with 0.1 MOI of Z1, Z7 (G11), or PBS as control and incubated for 3 days. The confocal images showed that ZIKV E protein was detected in Z1-infected cells starting from D2 p.i., while Z7-infected cells started to produce a lower level of the protein from D3 p.i. compared with Z1-infected cells (Fig. 3B). Consistently, the FFA results also showed that Z7 took a longer time (4 days) than Z1 (3 days) to develop the ZIKV E positive immunofoci (Fig. 3C). In addition, in the plaque-forming assay, Z7 took a prolonged time (5 days) compared to Z1 and ZIKV (strain PRVABC59, 4 days) to develop visible plaques. Moreover, Z7 developed smaller plaques by size than Z1 and ZIKV (strain PRVABC59) on D5 p.i. (Fig. 3D, E). These results collectively demonstrate that Z7 exhibits attenuated pathogenicity in vitro compared to Z1.

**Fig. 4:**
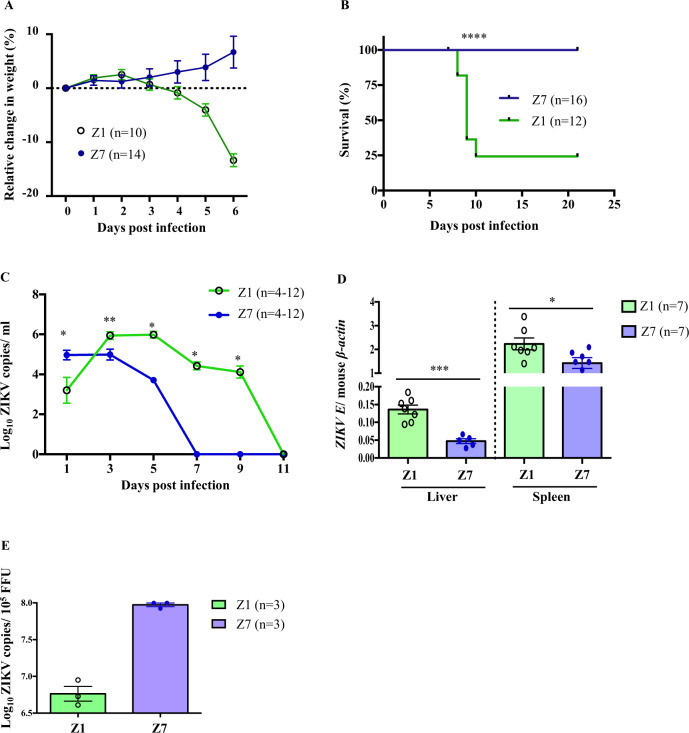
Z7 exhibits attenuated infectivity in *Ifnar1*^−/−^ mice. Four-week-old *Ifnar1*^−/−^ mice were inoculated with 1 × 10^5^ FFU of Z1, or Z7 (G10) through footpad. **A** Relative changes in body weight. **B** Survival curves. Mice were monitored daily for 21 days to determine survival. **C** Viremia of Z1 and Z7 infected mice. Mice were bled every-alternate day from D1 to D11 p.i., and the viremia was determinized by measuring the ZIKV genome copies (*ZIKV E*) by qRT-PCR. **D** Viral load in the liver and spleen tissues. Z1 or Z7 (G10)-infected mice were sacrificed on D3 p.i. to harvest the livers and spleens. The viral burden was measured by qRT-PCR and expressed as the ratio of the copy numbers of *ZIKV E* to mouse *β-actin*. **E** Viral RNA was extracted from the viral stocks containing 1 × 10^5^ FFU of Z1 or Z7 (G10). The *ZIKV E* genome copies were qualified by qRT-PCR. Data were compared with a log-rank test (**B**), Mann–Whitney U tests (**C**, **E**), a two-tailed Student’s *t*-test (**D**) and presented as mean ± s.e.m. with *p < 0.05, **p < 0.01, ***p < 0.001, and ****p < 0.0001.

To evaluate the pathogenicity of Z7 in a mouse model, we infected 4-week-old, sex-matched type I interferon (IFN) receptor A deficient (*Ifnar1*^−/−^) mice (in C57BL/6J background) with 1 × 10^5^ focus-forming units (FFU) of Z7 (G10) or Z1 via a footpad inoculation, which partially mimics mosquito transmission^39–43^. After infection, Z1-infected control mice began to lose body weight from D4 p.i. (Fig. 4A), death occurred from D8 p.i., and 68% of the mice died by the end of the experiment (D21 p.i., Fig. 4B). On the contrary, Z7 infected mice did not show any signs of sickness but instead continuously gained body weight, and 100% of the mice survived by the end of the experiment (Fig. 4A, B). To further evaluate the replication of Z7 in mice, we infected separate groups of *Ifnar1*^−/−^ mice with Z1 or Z7 as above and measured the *ZIKV E* gene replication in the blood, spleen, and liver by qRT-PCR. Consistent with the body weight and the survival results, Z7-infected mice generated a significantly lower viremia than Z1-infected animals from D3 to D9 p.i., and the *ZIKV E* gene in Z7-infected blood samples became undetectable or below the pre-set qRT-PCR detection limit (Cq = 38) after D7 p.i. (Fig. 4C). Similarly, Z7-infected mice also had reduced viral loads compared to Z1-infected mice in the liver and spleen tissues (Fig. 4D). Interestingly, significantly more ZIKV genome copies were detected in Z7-infected mice blood than that in Z1-infected mice blood samples on D1 p.i. (Fig. 4C). We hypothesized that there might be more non-infectious Z7 viral particles than Z1 in the inoculums. To test this, we pretreated 1 × 10^5^ FFU of Z1 and Z7 viral stocks with RNase to remove any cellular and viral RNA that is free of the viral particles, then extracted viral RNA from the viral nucleocapsids. ZIKV genome copies were quantified by measuring *ZIKV E* with qRT-PCR. The results showed 15-fold more ZIKV genome RNA copies in Z7 than Z1 within 1 × 10^5^ FFU of the viral stock samples (Fig. 4E). These results further indicated that Z7 possessed weakened infectivity and may produce a large amount of non-infectious viral particles, which could serve as a great source of immunogens to induce anti-ZIKV immunity without causing disease. In summary, both in vitro and in vivo results strongly suggest that Z7 exhibits attenuated pathogenicity compared to its parental WT strain Z1.

### Z7 induces robust humoral and cellular immune responses in *Ifnar1*^−/−^ mice

**Fig. 5:**
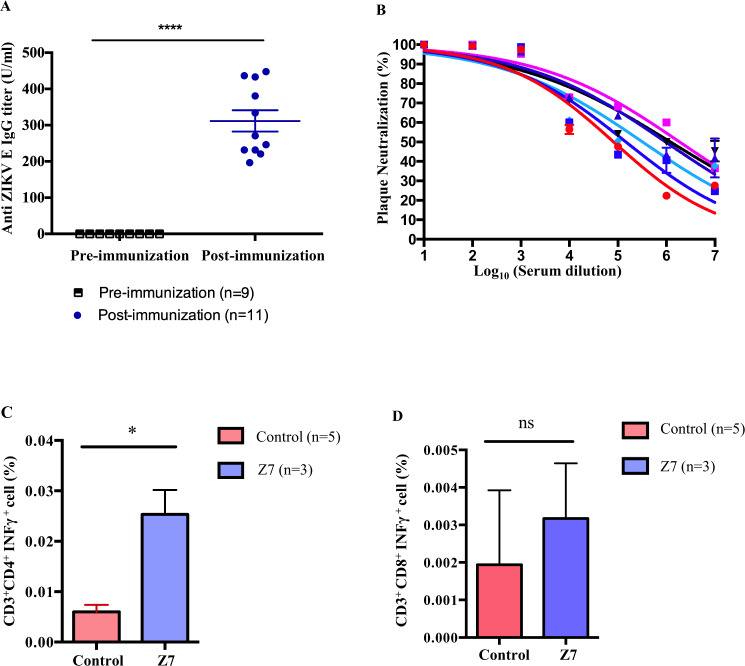
Z7 induces robust IgG and T-cell responses in mice. **A** Anti-ZIKV E IgG response of Z7 immunization. Four-week-old *Ifnar1*^−/−^ mice were immunized with 1 × 10^5^ FFU of Z7 (G10) via footpad. Anti-ZIKV E IgG was measured in the plasma samples from D0 (pre-immunization) and D24 (post-immunization) by ELISA. **B** PRNT. Six 4-week-old *Ifnar1*^−/−^ mice were immunized with 2 × 10^4^ FFU of Z7 (G11) via footpad. On D24 p.i., plasma samples were collected. The ZIKV neutralizing capacity of the plasma from each mouse was determined by PRNT. **C**, **D** Cellular immune responses of Z7 immunization. Seven-week-old *Ifnar1*^−/−^ mice were immunized with 1 × 10^5^ FFU of Z7 (G10) or PBS (control) via footpad. On D8 p.i., splenocytes were collected and re-stimulated with Z1 ex vivo for 24 h. The IFN-γ producing (C) CD4^+^ and (**D**) CD8^+^ T cells were measured by flow cytometry. Data were compared with a two-tailed Student’s *t*-test (**A**), Mann–Whitney U tests (**C**, **D**) and presented as mean ± s.e.m. with *p < 0.05, and ****p < 0.0001.

Since Z7 was generated by modifying the 5′ UTR of a non-epidemic ZIKV strain (FSS13025), and all the structural and non-structural genes remain untouched, we hypothesized that Z7 could induce robust humoral and cellular immune responses. To test this, we inoculated 4-week-old, sex-matched *Ifnar1*^−/−^ mice with 1 × 10^5^ FFU of Z7 via footpad. The blood samples were collected on D0 (pre-immunization) and D24 post-immunization. The levels of anti-ZIKV E IgG in the plasma were measured by ELISA. The ELISA results indicated that Z7 induced a high titer of anti-ZIKV E IgG (mean value = 311.7 U/ml) on D24 p.i. (Fig. 5A). To measure if Z7-induced antibodies could efficiently neutralize the epidemic ZIKV (strain PRVABC59) in vitro, we performed the plaque reduction neutralization test (PRNT). The range of the PRNT_50_ value of Z7-induced neutralizing antibody was between 10^4.9^ to 10^6.2^ (Fig. 5B). These results further suggest that Z7 immunization induces robust anti-ZIKV neutralizing antibody responses.

To measure the cellular immune responses, we immunized 7-week-old *Ifnar1*^−/−^ mice with 1 × 10^5^ FFU of Z7 or PBS as control via footpad and collected the spleens on D8 p.i. The splenocytes were re-stimulated with 0.1 MOI of Z1 ex vivo for 24 h to induce ZIKV-specific T-cell responses; the IFN-γ-producing CD4^+^ and CD8^+^ T cells were measured using flow cytometry. The results showed that the Z7-immunized mice induced approximately 3-fold higher IFN-γ response in CD4^+^ T cells than the control group (Fig. 5C and Supplementary Fig. 3). However, there was no difference in IFN-γ producing CD8^+^ T cells between the Z7-immunized group and the control group (Fig. 5D and Supplementary Fig. 3). Thus, these results indicate that Z7 immunization can induce strong humoral and cellular immune responses in *Ifnar1*^−/−^ mice.

### Z7 induces sterilizing immunity against ZIKV infection in *Ifnar1*^−/−^ mice

**Fig. 6:**
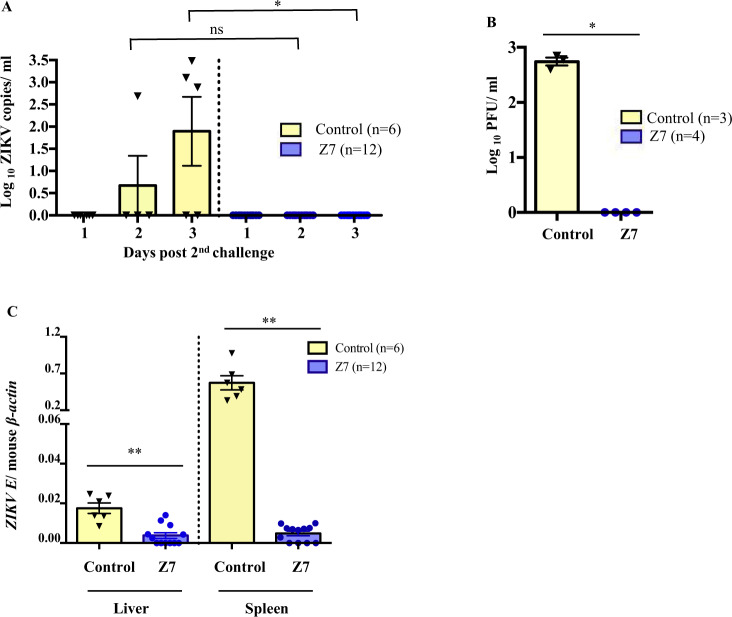
Z7 induces sterilizing immunity in *Ifnar1*^−/−^ mice against an epidemic ZIKV strain infection. Four-week-old *Ifnar1*^−/−^ mice were inoculated with 1 × 10^5^ FFU of Z7 (G10) or PBS as control via footpad. On D42 p.i., the mice were challenged with 1 × 10^5^ PFU of ZIKV (strain PRVABC59) via footpad. **A**, **B** Viremia. Mice were bled on D1 to D3 post-challenge (p.c.), and viremia was measured by (**A**) qRT-PCR on D1 to D3 p.c. and (**B**) plaque assay on D3 p.c. samples. **C** Viral load in the liver and spleen tissues. Z1 or Z7-infected mice were sacrificed on D3 p.i. to harvest the liver and spleen. The viral load in the tissues was measured by qRT-PCR and expressed as the ratio of *ZIKV E* to mouse *β-actin*. Data were compared with Mann–Whitney U tests (**A**, **B**), a two-tailed Student’s *t*-test (**C**) and presented as mean ± s.e.m. with *p < 0.05, and **p < 0.01.

To evaluate if Z7-induced immunity can protect mice from epidemic ZIKV infection, we immunized 4-week-old *Ifnar1*^−/−^ mice with either 1 × 10^5^ FFU of Z7 (G10) or PBS as control via footpad inoculation. On D42 post-immunization, we challenged these mice through a footpad with 1 × 10^5^ plaque forming unit (PFU) of ZIKV (strain PRVABC59). The blood samples were collected on D1 to D3 post the challenge (p.c.) to measure the viremia by quantifying *ZIKV E* gene copies by qRT-PCR. There were no detectable *ZIKV E* genes in the blood samples of Z7-immunized mice at any time point, whereas they were detected in the samples of the control group on D2 and D3 p.c. (Fig. 6A). To confirm the qRT-PCR results, we also measured the infectious viral particles in the blood samples collected on D3 p.c. by plaque-forming assay. Similar to qRT-PCR, no plaque was developed in the blood samples of the Z7-immunized mice (Fig. 6B). To measure the viral load in the peripheral tissue after the challenge with the epidemic ZIKV infection, we sacrificed some mice on D3 p.c. and collected the liver and spleen to measure the viral load by qRT-PCR. Consistent with the viremia, the *ZIKV E* genes in the tissue samples of Z7-immunized mice were either not detectable or below the set qRT-PCR detection limit (Cq = 38). The control mice had significantly more *ZIKV E* genes in both liver and spleen samples (Fig. 6C). Thus, these results suggest that a single dose of Z7 immunization can induce sterilizing immunity to protect mice against the epidemic ZIKV strain infection in the *Ifnar1*^−/−^ mouse model.

### Adoptive transfer of Z7-immunized plasma protects *Ifnar1*^−/−^ mice from ZIKV infection

**Fig. 7:**
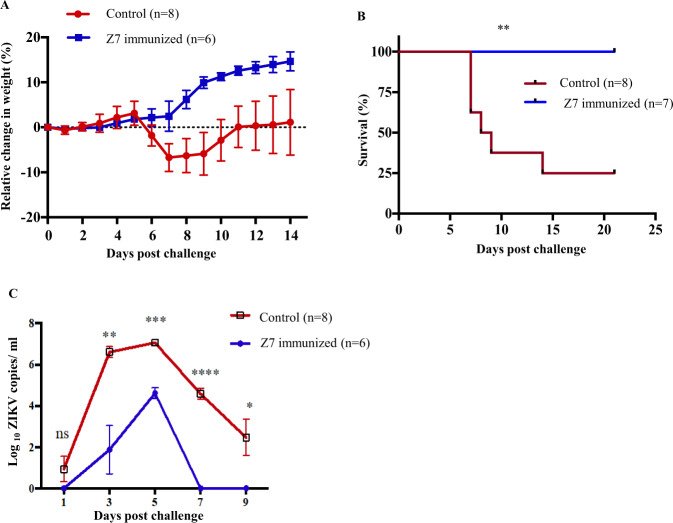
Z7 immunized plasma protects *Ifnar1*^−/−^ mice from ZIKV infection. Five-week-old *Ifnar1*^−/−^ mice were infused with 100 µl of plasma collected from Z7 (G10) immunized or control mice via retro-orbital injection. After 24 h, the mice were challenged with 1 × 10^5^ PFU of ZIKV (strain PRVABC59) via footpad inoculation. **A** Relative changes in body weight. **B** Survival curves. Mice were monitored daily for 21 days to determine the survival. **C** Viremia. Mice were bled every-alternate day from D1 to D9 p.i., and viremia was determinized by measuring the *ZIKV E* copies by qRT-PCR. Data were compared with a log-rank test (**B**), a two-tailed Student’s *t*-test (**C**) and presented as mean ± s.e.m. with *p < 0.05, **p < 0.01, ***p < 0.001, and ****p < 0.0001.

To evaluate if the plasma collected from the Z7-immunized mice protects against ZIKV infection, we adoptively transferred 100 µl of plasma collected from either Z7-immunized mice or mock-infected mice as control via retro-orbital injection in 5-week-old *Ifnar1*^−/−^ mice. The next day, we challenged both groups with 1 × 10^5^ PFU of ZIKV (strain PRVABC59) via footpad and monitored body weight changes up to D15 p.i. The control group started to lose weight from D5 p.i. until D7 p.i. followed by gaining weight from D8 p.i. until the end of the experiment (Fig. 7A). On the contrary, the mice which received the Z7-immunized plasma did not show any signs of sickness and gained body weight continuously. To measure survival, we adoptively transferred 100 µl of Z7-immunized plasma or PBS as control via retro-orbital injection in 4-week-old *Ifnar1*^−/−^ mice and challenged them with 1 × 10^5^ PFU of ZIKV (strain PRVABC59) via footpad. The control group started to die from D7 p.i. and 75% of the mice died before the end of the experiment (D21 p.i.); in contrast, all of the Z7 plasma-infused animals survived (Fig. 7B). To characterize the protective effects of Z7-immunized plasma, we did a separate adoptive transfer study in a group of 5-week-old *Ifnar1*^−/−^ mice and collected blood from D1 to D9 p.i. on every other day to measure the *ZIKV E* gene by qRT-PCR. The results showed that the control mice generated greater levels of the viral RNA than the mice who received the Z7 plasma infusion at all time points except D1 p.i. (Fig. 7C). Thus, the adoptive transfer study suggested that a single dose of plasma collected from Z7-immunized mice is sufficient to significantly inhibit ZIKV replication, rendering 100% survival in *Ifnar1*^−/−^ mice.

## Discussion

The current ZIKV vaccine candidates belong to three general categories: (a) inactivated vaccines^44,45^, (b) subunit vaccines including DNA, viral vector^46^, mRNA vaccines^38,47–50^, and (c) live-attenuated vaccines^43,51^. The safety and immunogenicity of the whole formalin-inactivated virus vaccines have been demonstrated in phase I clinical trials^19,52^. However, the inactivated vaccines may not generate robust T-cell immunity and usually require multiple doses and periodic boosters. ZIKV subunit vaccine candidates using virus-like particles, peptides, E protein, or non-replicating viral vectors to deliver structural protein genes may also have similar shortcomings to inactivated vaccines^53,54^. DNA and mRNA vaccines can usually generate both antibody and T-cell-based immunity. However, DNA vaccines may have the risk of triggering autoimmune diseases by eliciting anti-DNA antibody production and causing insertional mutagenesis^55^, and mRNA vaccines are not stable and may trigger unnecessary immune responses^56^. In contrast, live-attenuated vaccines can induce robust antibody and T-cell responses with a single dose of immunization. In addition, live-attenuated vaccines are cost-effective in both manufacturing and transportation. The successful development of live attenuated vaccines against yellow fever virus and Japanese encephalitis virus has proven that live attenuated viruses can be safe and effective antiviral vaccines.

A live-attenuated vaccine candidate for dengue virus (DENV) was developed by modifying the 3′ UTR region. Deletion of 30-nts within the 3′ UTR was shown to attenuate DENV in cell culture and animal models^57^. Live-attenuated ZIKV strains have been developed by deleting 10, 20, and 30-nts of the 3′ UTR region in the ZIKV genome^43,58^. While there are general risk concerns for using them in immunocompromised individuals, live-attenuated vaccine candidates developed by deleting portions of the 3′ UTR region in the ZIKV genome of a pre-epidemic Cambodian strain, FSS13025, showed great safety profiles in both NHP and mice^19,43,58^. However, whether modifications of the 5′ UTR of a flavivirus by inserting nucleotides affect the viral infectivity, has not been previously reported.

Here, we generated mutant ZIKVs by inserting GC-rich sequences after the SLB region of the 5′ UTR of the ZIKV genome of the pre-pandemic Cambodian strain, FSS13025. The inserts of 38-nt (Z5) and 50-nt (Z7) result in the formation of new hairpin structures in the region of 5′ UTR. These new hairpin structures may interfere with the functions of 5′ UTR and inhibit viral genome replication and protein translation because the cellular translation enzymes depend on stem-loop structures to begin replication, and ribosomes might stall when engaged with additional stem-loop and hairpin structures^31,32^. Interestingly, the enlarged loop in the SLB hairpin structure resulting from the 18-nt insertion (Z3) might significantly interrupt the ZIKV genome processing and translation of the viral proteins and thus could become lethal to ZIKV; however, the detailed mechanisms by which 5′ UTR of flaviviruses regulates the viral genome replication and translation are warranted for further investigation. Based on the viability of the mutants, we selected Z7 to further characterize and evaluate its potential as a live-attenuated vaccine candidate. Compared to Z1, the WT control, the replication rate of Z7 was lower; however, through the continuous passaging in Vero cells, its titer gradually increased, indicating that Z7 has adapted fitness mutations by continuous replication. The whole genome sequencing of the mutants confirmed that the insert remained stable at the exact location even after 10 generations. In addition, two types of mutations in 1417 position (S1417A or S1417T) were identified in the NS2B protein. The NS2B protein couples with NS3 protease to cleave viral proteins and facilitate NS3 proteolytic activity, which contributes to the host cell apoptosis and neuropathogenesis^21,59^. It has been reported that mutations in NS2B significantly alter the interaction between NS2B and NS3, followed by a decrease in the NS3 protease activity and ZIKV replication^60^. Z7 acquired the S1417A or S1417T mutation in NS2B, which might also affect NS3 protease; however, the detailed roles of the point mutations need further investigation.

It has been well-documented that ZIKV NS5 protein binds to and degrades human signal transducer and activator of transcription (STAT) 2 proteins downstream of type I IFN receptor signaling, inhibiting the expression of IFN-stimulated genes and induction of innate antiviral responses. Consequently, ZIKV evades host defense mechanisms and can efficiently replicate in human cells. ZIKV NS5 protein cannot target murine STAT2 to evade IFN signaling, which hinders the use of WT mice in studying ZIKV-induced diseases^61–63^. Therefore, *Ifnar1*^−/−^ mice have been commonly used to study the pathogenesis of ZIKV and to test antiviral vaccines in vivo^3,5,43,64^. To characterize the infectivity profile of Z7 in vivo, we infected *Ifnar1*^−/−^ mice with Z7 (G10 or G11) or Z1 as a control. The relative change in body weight and the survival experiments indicated that Z7 did not cause any sign of sickness in *Ifnar1*^−/−^ mice, albeit lower levels of viral load in blood and the peripheral tissues could be detected in Z7-infected mice. These results thus confirmed that Z7 has an attenuated infectivity compared to its parental strain, Z1.

One of the advantages of developing the live attenuated viral vaccine by editing the UTR regions is that the remaining genome, including the entire immunogenic coding sequence, remains intact, thus inducing the identical profile of the immune responses as WT viruses. The Z7 ELISA and PRNT data suggested that a single dose of Z7 immunization could induce a high level of anti-ZIKV E IgG and total neutralizing antibodies. In line with this, the adoptive transfer of anti-Z7 serum could also protect the mice from losing body weight and dying due to the challenge of the epidemic ZIKV infection. In addition, compared to killed inactivated vaccines, the live attenuated vaccines replicate in host cells, thus facilitating viral peptides loading onto major histocompatibility complexes (MHC) for antigen presentation and producing strong CD4^+^ and CD8^+^ T-cell-mediated immune response. As a live attenuated ZIKV vaccine candidate, Z7 immunization could induce higher IFN-γ producing CD4^+^ and CD8^+^ T cells compared to the control. These results provide strong evidence that a single dose of Z7 immunization induces robust humoral and cellular immunity in the *Ifnar1*^−/−^ mouse model. Importantly, our results also suggest that mice pre-immunized with Z7 can be completely protected from the infection of the epidemic ZIKV in blood, livers, and spleens.

There are still several open-ended questions needed to be addressed in the future study, such as whether Z7 can be used during pregnancy to protect both the mother and fetus; whether Z7 can protect against sexually-transmitted ZIKV infection; whether Z7 is safe to be used in immunocompromised individuals; and whether Z7 can induce antibody-dependent enhancement reacting to other closely related flaviviruses, such as DENV or West Nile Virus. Despite these questions, Z7 shows great promise to be an effective vaccine candidate against ZIKV infection. A single dose of Z7 immunization can induce robust humoral and T-cell responses that protect mice from ZIKV infection. In addition, our results suggest that modifying the 5′ UTR can lead to a live-attenuated vaccine candidate for ZIKV, and this novel strategy may be potentially applied to other flaviviruses.

## Methods

### Ethics statement and biosafety

All the animal experimental procedures used in this study were reviewed and approved by the Institutional Animal Care and Use Committees (IACUC) at the University of Southern Mississippi (USM) under the IACUC protocol # 16031002. The experiments involving live ZIKV were performed by certified personnel in the Bio-safety level 2 (BSL-2, cell culture) and BSL-3 (animal) laboratories, following the biosafety protocols approved by the USM Institutional Biosafety Committee.

### Cells and viruses

The in vitro experiments were performed on Vero (ATCC CCL-81) or HEK-293 cells (ATCC CRL-1573) and maintained in Dulbecco’s modified Eagle’s medium (DMEM, Life Technologies) supplemented by 1% Penicillin/Streptomycin (P/S, Gibco), and 10% Fetal Bovine Serum (FBS, Atlanta Biologicals). The cells were kept in an incubator at 37 °C with 5% CO_2_ and relative humidity of about 95%. The ZIKV Puerta Rico strain (PRVABC59, GenBank number KU501215) was obtained from B. Johnson (CDC Arbovirus Branch), propagated in Vero cells, and quantified by plaque assay. The ZIKV plasmids of the Cambodian strain (GenBank number KU955593.1) were generously provided by Dr. Pei-yong Shi at the University of Texas Medical Branch (UTMB) and used for generating Z1.

### Plasmid construction, transmission, and virus collection

A pFLZIKV plasmid containing the Cambodian wild type ZIKV sequence FSS13025 (GenBank number KU955593.1) with the pACYC177 backbone and a pCC1BAC-PRV plasmid were a gift from Dr. P-Y Shi^65,66^. The cloning strategy involved adding a CMV promoter to the 5′ UTR of WT Z1 sequence in order to generate the FSS13025 viral RNA and changing the backbone from pACYC177 to pCC1BAC to facilitate plasmid propagation in *E. coli*. Three overlapping DNA fragments (F1—pCC1BAC backbone, F2—CMV-5′UTR-C-prM-E, and F3—E-NS1-NS5-3’UTR-HDVr-SV40 poly[A] signal) were generated by PCR using the DNA polymerase Q5 (NEB), and they were then assembled into the Z1 plasmid by NEBuilder HiFi DNA Assembly (NEB) into the full size 19,482 bp plasmid containing the Z1. The major portion of the viral sequence (E-NS1-NS5-3’UTR-HDVr) was first cloned into a plasmid Fb to add SV40 poly(A) signal by in vivo cloning^35^. Fb was then used as the template to generate F3. Detailed information is shown in Table 2.

**Table 2:** DNA fragments generated by PCR for ZIKV WT cloning to yield Z1.

DNA name	Template	Primer name	Primer sequence
F1	pCC1BAC-PRV	P1f P1r	AGCTTGGCGTAATCATGGTCATAGCTG GATCGGCACGTAAGAGGGGACTTCCATTGTTCATTCCACG
F2	F21/F22	P2f P2r	AATGAACAATGGAAGTCCCCTCTTACGTGCCGATCAAGTC GGGTTAGCGGTTATCAACCTCCCAACT
F21	pcDNA3 Kan	P2f P21r	AATGAACAATGGAAGTCCCCTCTTACGTGCCGATCAAGTC CGGTTCACTAAACGAGCTCTGCTTATATAGACCTCCCA
F22	pFLZIKV	P22f P2r	GCAGAGCTCGTTTAGTGAACCGAGTTGTTGATCTGTGTGA GGGTTAGCGGTTATCAACCTCCCAACT
F3	Fb	P3f P3r	GGACCTTGCAAGGTTCCAGCTCAGA GTGTGAAATACCCCGAACCCATGATCCT

Vero cells were plated at 2.5 × 10^5^ cells/well in 6-well plates and incubated for 24 h at 37 °C with 5% CO_2_. Z1, Z3, Z5, and Z7 plasmids (500 ng) were transfected with Lipofectamine 3000 reagent (ThermoFisher Scientific) according to the user’s manual. The Vero cells were allowed to incubate at 37 °C with 5% CO_2_ for 5 days to collect the cell culture supernatant containing the WT (Z1) and mutant (Z3, Z5, and Z7) viruses. To continuously pass the Z7 on Vero cells, 150 µl of the virus-containing medium from the previous passage was inoculated into 2.5 × 10^5^ Vero cells in 6-well plates for 5 days. The culture supernatant from each generation was collected and stored in a −80 °C freezer. The viral titer of Z1, Z5, and Z7 (G10 and G11) were determined by FFA.

### Mice and animal study

The breeding pairs of *Ifnar1*^−/−^ mice in C57BL/6J background (Stock # 028288) were purchased from the Jackson Laboratory. The breeding pairs and pups were kept in a clean room, and the infection experiments were performed in an animal BSL-3 lab at USM. For ZIKV infection, four-week-old *Ifnar1*^−/−^ mice were subcutaneously injected on the ventral side of the left hind footpad with 1 × 10^5^ FFU of Z1 or Z7 (G10) in phosphate buffer saline (PBS) on D0. The body weight of the infected mice was measured daily for 8 days p.i. Blood samples were collected on alternate days from D1 to D9 p.i. in 0.5 M Ethylenediaminetetraacetic acid (EDTA) from the retro-orbital sinus under isoflurane anesthesia, and the level of *ZIKV E* gene was measured by qRT-PCR. To measure the level of anti-ZIKV E IgG by ELISA (Alpha Diagnostic International), blood samples were collected on D0 and D24 p.i. to prepare plasma. On D42 p.i., the mice were challenged with 1 × 10^5^ PFU of ZIKV (strain PRVABC59), and blood was collected from D1 to D3 p.c., to measure the viremia by qRT-PCR and plaque assay. On D3 p.c., the mice were euthanized, and the liver and spleen were collected to measure the viral load by qRT-PCR. Data were normalized by mouse *β-actin* as a house-keeping gene.

### Quantitative reverse transcription polymerase chain reaction (qRT-PCR)

The total RNA was extracted from cell culture and tissue samples using TRI Reagent (Molecular Research Center, Inc). The RNA was converted into the first-strand complementary DNA (cDNA) using iSCRIPT^TM^ cDNA synthesis kit (Bio-Rad). The qRT-PCR assays were performed in a CFX Connect Real-Time System (Bio-Rad) using iTaq™ Universal Probes Supermix (Bio-Rad) for the detection of *ZIKV E* ^67,68^, and cellular *β-actin*^69^. ZIKV genome copies were calculated by measuring *ZIKV E* with qRT-PCR^39^.

### Viral growth kinetics

Vero or HEK-293 cells were plated at 5 × 10^5^ or 2.5 × 10^5^ cells/well respectively in 12-well plates and incubated overnight at 37 °C with 5% CO_2_. The cells were then infected with 0.1 MOI (Vero cell) or 0.01 MOI (HEK-293 cell) of Z1 or Z7 (G11) and incubated for 1 h. The medium was replaced with 1 ml of DMEM supplemented with 10% FBS, and 1% P/S, and incubated for 6 days. The culture supernatants containing either Z1 or Z7 were collected daily from D1 to D6. The viral titers of Z1 and Z7 were determined by FFA.

### Cytopathic effect assay (CPE)

Vero cells were plated at 5 × 10^4^ cells/well in 12-well plates and incubated overnight at 37 °C with 5% CO_2_, then infected with 0.1 MOI of Z1 and Z7 (G11) and incubated for 1 h at 37 °C. The medium was then replaced with 1 ml of DMEM supplemented with 10% FBS and 1% P/S and incubated for 3 days. On D3 p.i., the cells were stained using LIVE/DEAD Cell Imaging Kit (488/570, ThermoFisher Scientific) according to the user’s manual. The images were taken using a Leica M165 FC microscope (Leica Microsystems).

### Immune fluorescence assay (IFA)

Vero cells were plated at 1.25 × 10^4^ cells/well in 24-well glass-bottom plates and incubated overnight at 37 °C with 5% CO_2_. The cells were then infected with 0.1 MOI of Z1, Z7 (G11), or PBS as a control and incubated for 1 h at 37 °C. After incubation, the cell medium was replaced with 500 µl of DMEM, and the cells were incubated at 37 °C for 3 days. The cells were fixed with 250 µl of 4% Para-formaldehyde solution (PFA) in PBS for 15 min at room temperature (RT), followed by 2× washes with PBS. The cells were permeabilized with 250 µl of 0.1% Triton-X for 20 min at RT followed by 2× washes with PBS, then blocked with 500 µl of 5% skim milk in 0.1% PBST (PBS with Tween-20) for 1 h at 4 °C. The cells were then probed with the mouse anti-flavivirus glycoprotein E IgG antibody (4G2, in-house produced from the hybridoma D1-4G2-4-15 HB-112, ATCC), diluted with 5% skim milk in 1:50 ratio, 200 µl/well, incubated overnight at 4 °C followed by two 5-min washes with 0.1% PBST. The cells were then probed with goat anti-mouse IgG conjugated with Alexa Fluor 488 (Jackson ImmunoResearch Laboratories, catalog # 112-545-003), diluted with 5% skim milk in 1:100 ratio, 200 µl/well, at 4 °C in the dark for 2 h, followed by two 5-min washes with 0.1% PBST. The nuclei of the cells were stained with 600 nM DAPI solution at RT for 10 min. The images were taken using a Stellaris STED confocal microscope (Leica Microsystems).

### Focus forming assay (FFA)

Vero cells were plated at 5 × 10^5^ cells/well in 12-well plates and incubated overnight at 37 °C with 5% CO_2_. The cells were inoculated with serially diluted viruses Z1 or Z7 (G10 or G11) and incubated for 2 h at 37 °C. Then the medium was replaced with 1 ml/well of 1 x Opti MEM GlutaMAX (Gibco) medium supplemented with 1% Methylcellulose (Sigma), 10% FBS, and 1% P/S, and incubated for 3 days (Z1) or 4 days (Z7). After the incubation, the overlay medium was removed and washed gently with PBS. The plates were fixed with 4% PFA for 15 min, permeabilized with 0.1% Triton-X for 20 min at RT, and blocked with 5% skim milk for 1 h. The cells were then probed with 4G2 antibody diluted with 5% skim milk in 1:50 ratio, incubated at 4 °C overnight in the dark followed by two 5-min washes with 0.1% PBST. The cells were then probed with goat anti-mouse IgG conjugated with horseradish peroxidase (HRP) (Abcam, catalog # ab97023), diluted with 5% skim milk in 1:500 ratio, incubated at 4 °C in the dark for 2 h, followed by two 5-min washes with 0.1% PBST, and air dried for 20 min at RT. The Immuno-positive foci were developed with TrueBlue peroxidase substrate (KPL, Sera care)^70^.

### Plaque assay

Vero cells were plated at 6 × 10^5^ cells/well in 6-well plates and incubated overnight at 37 °C with 5% CO_2_. One ml of serially diluted ZIKV (strain PRVABC59), Z1, or Z7 were incubated with the Vero cells monolayer for 1 h at 37 °C. Then the virus-containing medium was replaced with DMEM overlay medium containing 1% SeaPlaque Agarose (Lonza), 10% FBS, and 1% P/S and incubated for 5 days. The plaques were stained with 0.1% Neutral red solution (Sigma) and counted^68^. The plaque size on D5 p.i., was measured by ImageJ software (National Institute of Health) with the ViralPlaque add-in^71^ and expressed as area in pixel^2^.

### Plaque reduction neutralization test (PRNT)

Vero cells were plated at 6 × 10^5^ cells/well in 6-well plates and incubated overnight. The plasma samples were incubated at 56 °C for 30 min to inactivate the complements, serially diluted from 10^1^ to 10^7^ folds with pre-warm, serum-free DMEM. The diluted samples were mixed with 100 PFUs of ZIKV (strain PRVABC59) and incubated at 37 °C for 1 h. The virus-plasma mixtures were applied onto Vero cells and incubated for 1 h. Then the virus-containing medium was replaced with DMEM overlay medium containing 1% SeaPlaque Agarose (Lonza), 10% FBS, and 1% P/S and incubated for 4 days. The plaques were stained with 0.1% Neutral red solution (Sigma) and counted^68^. Then the PRNT_50_ values were calculated with GraphPad Prism software (version 7.0)^72^.

### Flow cytometry

Seven-week-old *Ifnar1*^−/−^ mice were infected with 1 × 10^5^ FFU of Z7 (G10) or PBS (control). Mice were euthanized on D8 p.i. to collect splenocytes. The splenocytes (3 × 10^6^) were plated in 6-well plates and re-stimulated with 0.1 MOI of Z1 for 24 h at 37 °C. During the final 8 h of the stimulation, Brefeldin A solution (BD Bioscience) was added in 1:1000 to block cytokine secretion. Cells were then stained with antibodies against mouse CD3 (FITC-conjugated, 0.25 µg/test, 1:200, BD Biosciences, catalog # 555274), CD4 (PerCp Cy 5.5-conjugated, 0.2 µg/test, 1:100, BD Biosciences, catalog # 550954), and CD8 (APC-conjugated, 0.2 µg/test, 1:100, BD Biosciences, catalog # 553035). Cells were fixed with 2% PFA overnight and permeabilized with 1 x Permeabilization buffer. Cells were then intracellularly stained with PE-Cy7 conjugated anti-IFNγ (0.2 µg/test, 1:100, BD Biosciences, catalog # 557649). Data were acquired using BD LSRFortessa^TM^ Cell Analyzer (BD Biosciences) and analyzed with FlowJo (v10.8.0.) software.

### Transmission electron microscopy

Z1 and Z7 (G9) virus samples were centrifuged at 2655 × *g* for 5 min at 4 °C to remove cell debris. Viral samples were initially added to the top of 20% sucrose cushion in Polyallomer ultracentrifuge tubes (Beckman Coulter Life Sciences) and were centrifuged by Optima XPN-80 Ultracentrifuge (REVCO) at 58,563 × *g* for 2 h at 4 °C. The pellets were then resuspended and fixed with 4% glutaraldehyde solution (Sigma). The virus samples were negatively stained with 2% Uranyl acetate solution and visualized under a Transmission Electron Microscope (TEM)^38^. The TEM images of Z1 and Z7 were taken by JEOL JEM-1400 120 kV TEM machine in the Shared Instrumental Facility of Louisiana State University (Baton Rouge, LA).

### Next-generation sequencing

Vero cells were plated at 2 × 10^5^ cells/well in 6-well plates, incubated overnight at 37 °C with 5% CO_2_, and infected with 0.5 MOI of Z7 (G8-G10) or Z1 for 48 h. Total RNA was extracted using RNeasy Mini Kit (Qiagen) for RNA sequencing using the Illumina platform (Psomagen). The paired reads were processed to generate de novo sequences which were aligned to the ZIKV sequence (Cambodian strain, FSS13025, GenBank number KU955593.1).

### Statistical analysis

Data were compared using Mann–Whitney U test, log-rank test, or two-tailed Student’s *t*-test with GraphPad Prism software (version 7.0), whichever was applicable.

### Reporting summary

Further information on research design is available in the Nature Research Reporting Summary linked to this article.

## Supplementary information

Supplemenatry materials

REPORTING SUMMARY

## Data Availability

The data supporting the findings of this study are available from the corresponding author.
