# Supplementary material for: An effective live-attenuated Zika vaccine candidate with a modified 5′ untranslated region
Source: NPJ Vaccines. 2023 Apr 1;8:50. doi: 10.1038/s41541-023-00650-w (PMC10066991; doi:10.1038/s41541-023-00650-w)
Supplement: Supplementary file 1 — Supplemenatry materials [file 41541_2023_650_MOESM1_ESM.pdf]

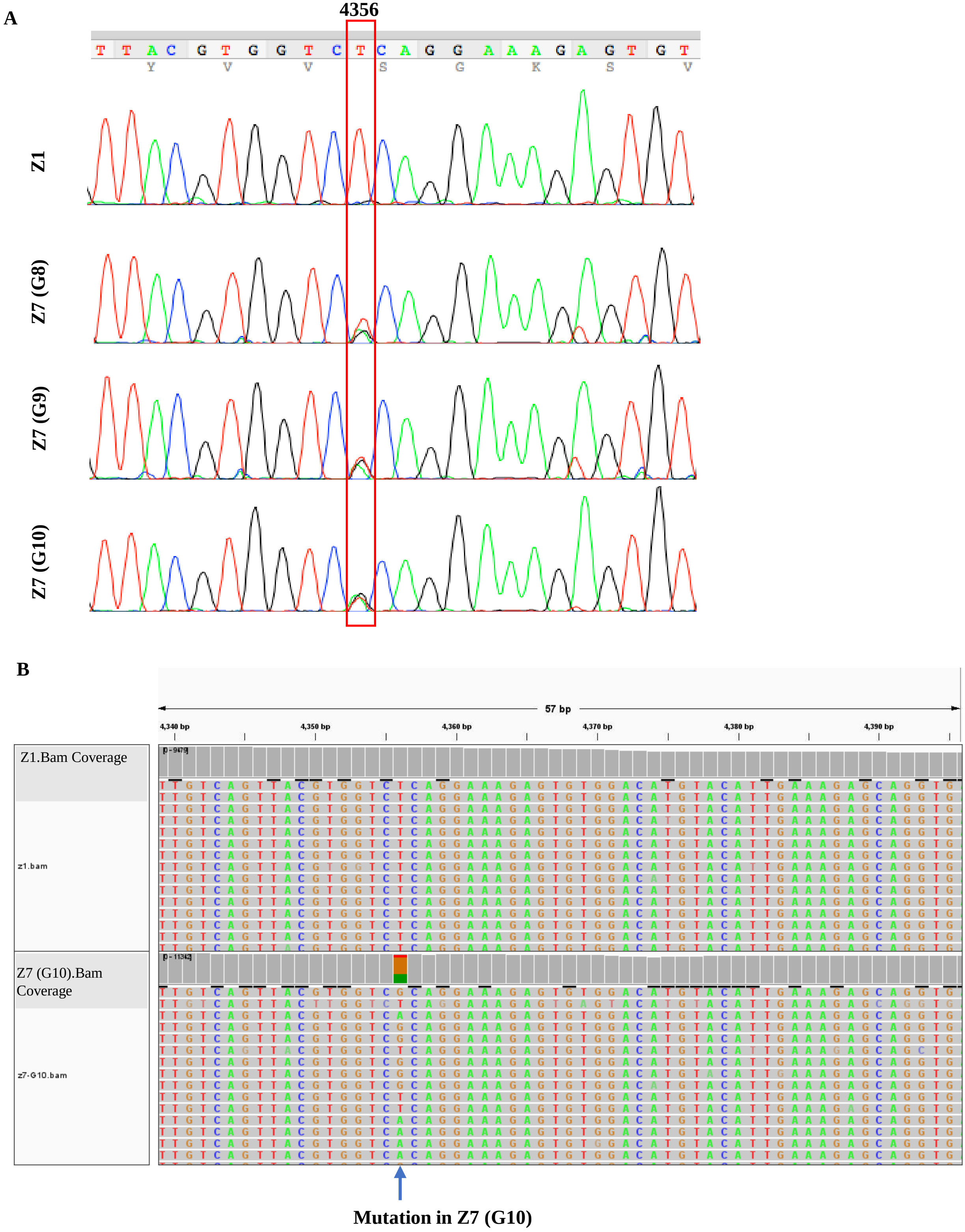

Supplementary Figure 1. Nucleic acid mutations in Z7. (A) DNA sequences of Z1 and Z7 (G8 to G10) generated by Sanger sequencing show the mutations in *NS2B*. The mutation position in nucleotide is 4356 (amino acid position 1417) of the ZIKV genome (Cambodian strain, FSS13025, GenBank number KU955593.1). (B) RNA sequences of Z1 and Z7 (G10) show the mutations (T [U]→G primary; or T [U]→A secondary). The bam sequences were generated by aligning the Fasta files against the Cambodian strain ZIKV sequence.

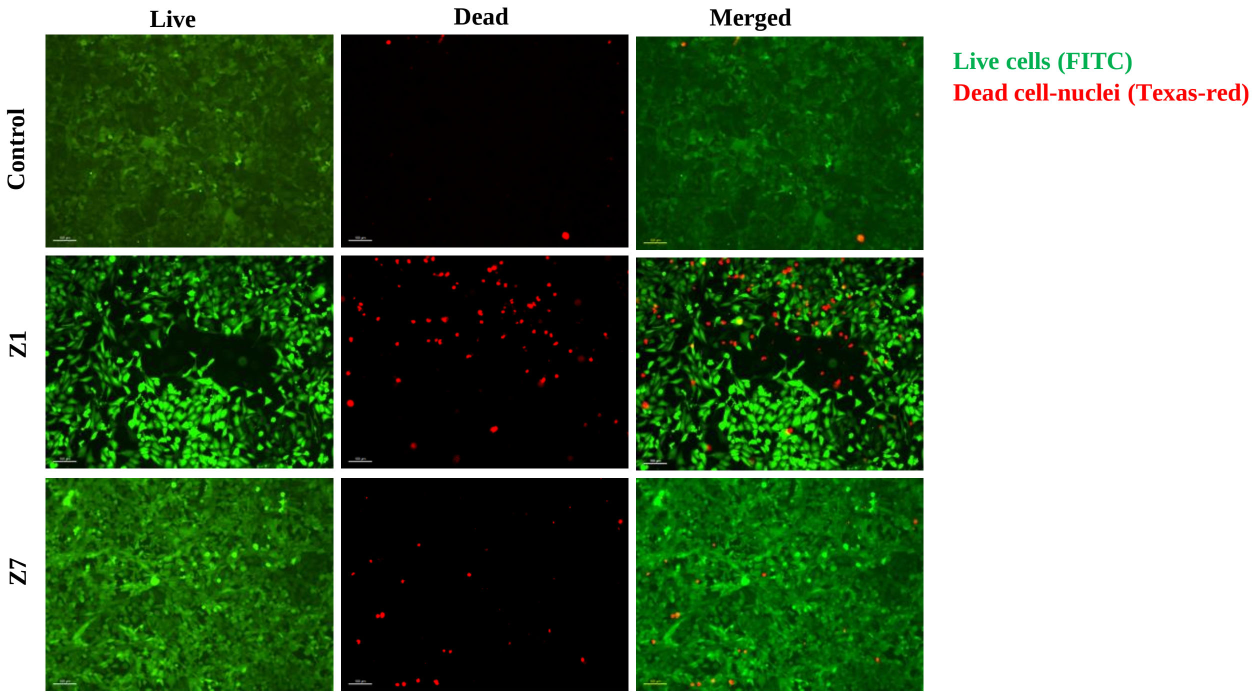

Supplementary Figure 2. The CPE of Z1 and Z7 in Vero cells on D4 p.i. Vero cells were inoculated with Z1, Z7 (G11), or PBS as a control and incubated for four days. The cells were stained with LIVE/DEAD Cell Imaging Kit, which stained the live cells with FITC as green and the dead cell nuclei with Texas-red as red. The images were taken at 10 x magnification (scale bar = 100 µm).

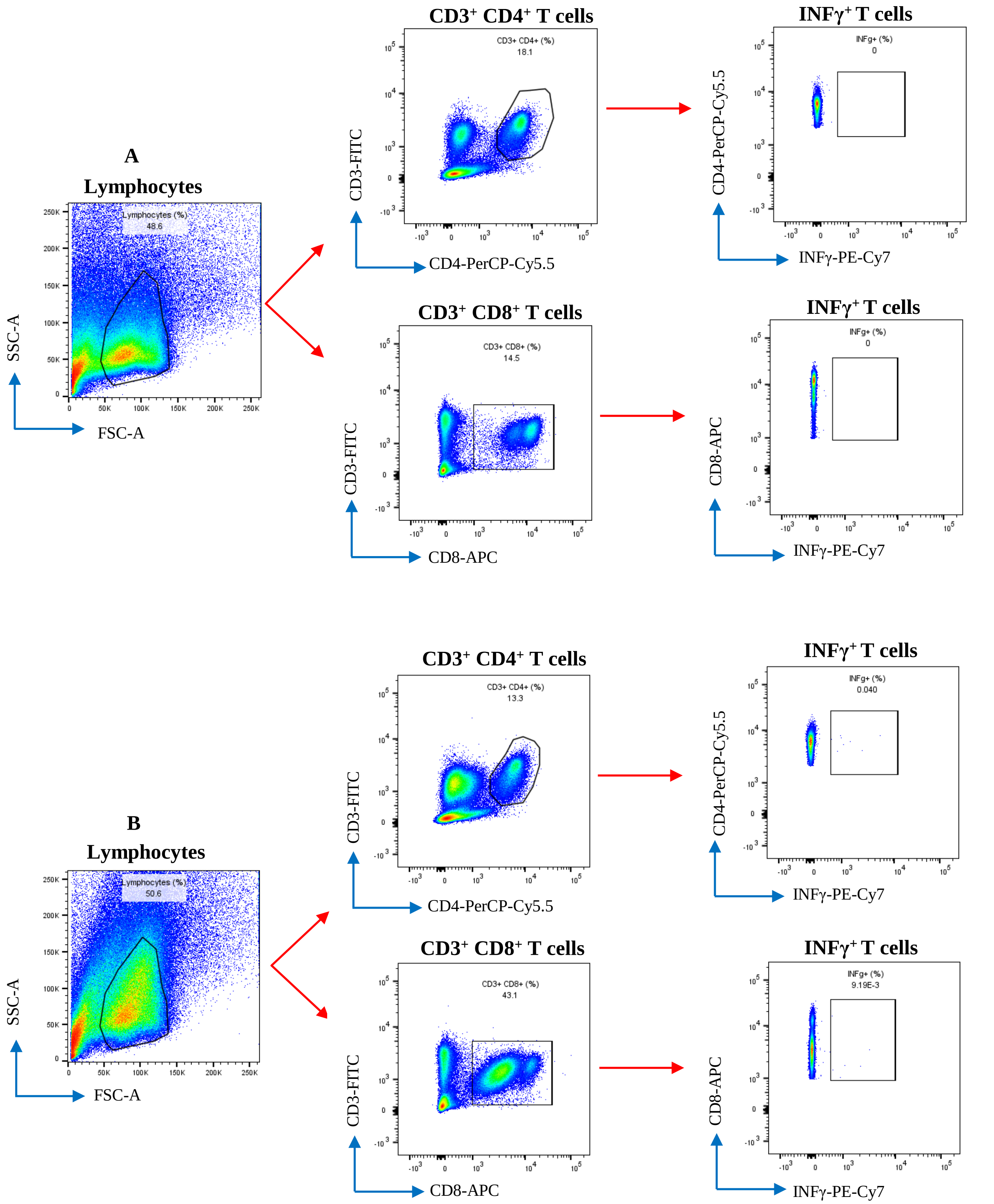

Supplementary Figure 3. Cell mediated immune response in Z7 immunization. Seven-week-old *Ifnar1*$^{-/-}$ mice were immunized with 1 x 10^5 FFU of Z7 (G10) or PBS (control) via footpad. On D8 p.i., splenocytes were collected, re-stimulated with Z1 ex vivo and analyzed by flow cytometry. Gating strategy used to detect the IFN-γ producing $CD4^+$ and $CD8^+$ T cells in (A) control and (B) Z7 immunized mice.
