# Supplementary material for: An effective live-attenuated Zika vaccine candidate with a modified 5′ untranslated region
Source: NPJ Vaccines. 2023 Apr 1;8:50. doi: 10.1038/s41541-023-00650-w (PMC10066991; doi:10.1038/s41541-023-00650-w)
Supplement: Supplementary file 2 — REPORTING SUMMARY [file 41541_2023_650_MOESM2_ESM.pdf]

nature portfolio

Corresponding author(s): Fengwei Bai

Last updated by author(s): 2023/03/10

Reporting Summary

Nature Portfolio wishes to improve the reproducibility of the work that we publish. This form provides structure for consistency and transparency in reporting. For further information on Nature Portfolio policies, see our Editorial Policies and the Editorial Policy Checklist.

Statistics

For all statistical analyses, confirm that the following items are present in the figure legend, table legend, main text, or Methods section.

n/a	Confirmed	
☐	☒	The exact sample size (*n*) for each experimental group/condition, given as a discrete number and unit of measurement
☐	☒	A statement on whether measurements were taken from distinct samples or whether the same sample was measured repeatedly
☐	☒	The statistical test(s) used AND whether they are one- or two-sided<br>*Only common tests should be described solely by name; describe more complex techniques in the Methods section.*
☒	☐	A description of all covariates tested
☐	☒	A description of any assumptions or corrections, such as tests of normality and adjustment for multiple comparisons
☐	☒	A full description of the statistical parameters including central tendency (e.g. means) or other basic estimates (e.g. regression coefficient) AND variation (e.g. standard deviation) or associated estimates of uncertainty (e.g. confidence intervals)
☐	☒	For null hypothesis testing, the test statistic (e.g. *F*, *t*, *r*) with confidence intervals, effect sizes, degrees of freedom and *P* value noted<br>*Give P values as exact values whenever suitable.*
☒	☐	For Bayesian analysis, information on the choice of priors and Markov chain Monte Carlo settings
☒	☐	For hierarchical and complex designs, identification of the appropriate level for tests and full reporting of outcomes
☒	☐	Estimates of effect sizes (e.g. Cohen's *d*, Pearson's *r*), indicating how they were calculated

Our web collection on statistics for biologists contains articles on many of the points above.

Software and code

Policy information about availability of computer code

Data collection	Fig. 3E plaque sizes were collected with Image J.
Data analysis	Data were analyzed with GraphPad Prism software (version 7.0)

For manuscripts utilizing custom algorithms or software that are central to the research but not yet described in published literature, software must be made available to editors and reviewers. We strongly encourage code deposition in a community repository (e.g. GitHub). See the Nature Portfolio guidelines for submitting code & software for further information.

Data

Policy information about availability of data

All manuscripts must include a data availability statement. This statement should provide the following information, where applicable:

- Accession codes, unique identifiers, or web links for publicly available datasets
- A description of any restrictions on data availability
- For clinical datasets or third party data, please ensure that the statement adheres to our policy

All the data will be available through publication or by request.

Human research participants

Policy information about [studies involving human research participants and Sex and Gender in Research.](#)

Reporting on sex and gender	N/A
Population characteristics	N/A
Recruitment	N/A
Ethics oversight	N/A

Note that full information on the approval of the study protocol must also be provided in the manuscript.

Field-specific reporting

Please select the one below that is the best fit for your research. If you are not sure, read the appropriate sections before making your selection.

☒ Life sciences ☐ Behavioural & social sciences ☐ Ecological, evolutionary & environmental sciences

For a reference copy of the document with all sections, see nature.com/documents/nr-reporting-summary-flat.pdf

Life sciences study design

All studies must disclose on these points even when the disclosure is negative.

Sample size	Sample sizes were determined by power calculation.
Data exclusions	No data excluded.
Replication	Most of the experiments have been repeated at least once. However, TEM images in Fig. 1E were only acquired in one experiment. These are representative images from more than 10 different images at different fields under a TEM.
Randomization	The sex, age-matched mice were randomly placed in either control or experimental groups.
Blinding	We used the blinding approach as much as possible during our experiments.

Reporting for specific materials, systems and methods

We require information from authors about some types of materials, experimental systems and methods used in many studies. Here, indicate whether each material, system or method listed is relevant to your study. If you are not sure if a list item applies to your research, read the appropriate section before selecting a response.

Materials & experimental systems

n/a	Involved in the study	
☐	☒	Antibodies
☐	☒	Eukaryotic cell lines
☒	☐	Palaeontology and archaeology
☐	☒	Animals and other organisms
☒	☐	Clinical data
☒	☐	Dual use research of concern

Methods

n/a	Involved in the study	
☒	☐	ChIP-seq
☐	☒	Flow cytometry
☒	☐	MRI-based neuroimaging

Antibodies

Antibodies used	4G2, in-house produced from the hybridoma D1-4G2-4-15 HB-112, ATCC; Goat anti-mouse IgG conjugated with Alexa Fluor 488 (Jackson ImmunoResearch Laboratories, catalog # 112-545-003), CD3 (FITC-conjugated, BD Biosciences, catalog # 555274), CD4 (PerCp Cy 5.5-conjugated, BD Biosciences, catalog # 550954), CD8 (APC-conjugated, BD Biosciences, catalog # 553035), PE-Cy7 conjugated anti-IFNγ (BD Biosciences, catalog # 557649), and goat anti-mouse IgG conjugated with horseradish peroxidase (HRP) (Abcam, catalog # ab97023).

Validation | 4G2 antibody was produced in-house, and we have tested it binds to West Nile virus, dengue virus, and Zika virus E proteins (PMID: 27441043, 33304351, 23580653; https://doi.org/10.3390/vaccines11020197). Validation of the other antibodies are described in the vendor's website.

Eukaryotic cell lines

Policy information about cell lines and Sex and Gender in Research

Cell line source(s)	All the cell lines used in this manuscript were purchased from ATCC.
Authentication	The cells were purchased from ATCC and were not further authenticated.
Mycoplasma contamination	The cells were purchased from ATCC and have not been tested for mycoplasma contamination.
Commonly misidentified lines (See ICLAC register)	N/A

Animals and other research organisms

Policy information about studies involving animals; ARRIVE guidelines recommended for reporting animal research, and Sex and Gender in Research

Laboratory animals	WT(C57BL/6J) and IFNAR1-/- Mice were purchased from the JAX lab.
Wild animals	N/A
Reporting on sex	We use sex- and age-matched mice for each experiment.
Field-collected samples	N/A
Ethics oversight	All the animal experimental procedures used in this study were reviewed and approved by the Institutional Animal Care and Use Committees (IACUC) at the University of Southern Mississippi (USM) under the IACUC protocol # 16031002.

Note that full information on the approval of the study protocol must also be provided in the manuscript.

Flow Cytometry

Plots

Confirm that:

- [ ] The axis labels state the marker and fluorochrome used (e.g. CD4-FITC).
- [ ] The axis scales are clearly visible. Include numbers along axes only for bottom left plot of group (a 'group' is an analysis of identical markers).
- [ ] All plots are contour plots with outliers or pseudocolor plots.
- [x] A numerical value for number of cells or percentage (with statistics) is provided.

Methodology

Sample preparation	Single cells were prepared from mouse spleens.
Instrument	Data were acquired using BD LSRFortessa.
Software	Data were analyzed with FlowJo (v10.8.0.) software.
Cell population abundance	N/A
Gating strategy	We plotted cells with FSC and SSC, single cells, CD3CD4IFNg positive, or CD3CD8IFNg positive cells.

- [x] Tick this box to confirm that a figure exemplifying the gating strategy is provided in the Supplementary Information.
